# Predictive Value of Procalcitonin and C-reactive Protein for Mortality in Live Donor Liver Transplant Recipients

**DOI:** 10.7759/cureus.100440

**Published:** 2025-12-30

**Authors:** Saraswathy S, Johns Shaji Mathew, Anila KN, Sreerenjini B, Georg Gutjahr, Shweta Mallick, Binoj ST, Unnikrishnan G, Dinesh Balakrishnan, Sudhindran S

**Affiliations:** 1 Department of Gastrointestinal Surgery and Solid Organ Transplantation, Amrita Institute of Medical Sciences and Research Center, Amrita Vishwa Vidyapeetham, Kochi, IND; 2 Department of Multi-Organ Transplant and Hepato-Pancreatico-Biliary (HPB) Surgery, Burjeel Medical City, Abu Dhabi, ARE; 3 Department of Mathematics, Amrita Institute of Medical Sciences and Research Center, Amrita Vishwa Vidyapeetham, Kochi, IND; 4 Department of Health Science Research Center, Amrita School of Medicine, Amrita Institute of Medical Sciences and Research Center, Amrita Vishwa Vidyapeetham, Kochi, IND

**Keywords:** c-reactive protein, live donor liver transplantation, post-operative, procalcitonin, sepsis

## Abstract

Background: Accurate biomarkers are crucial for the early diagnosis of sepsis after liver transplantation. This study aimed to evaluate the diagnostic accuracy of procalcitonin (PCT) and C-reactive protein (CRP) in predicting bacterial infections and mortality following living donor liver transplantation (LDLT).

Methodology: We prospectively analyzed all adult LDLT patients at a tertiary center between January 2021 and December 2022. CRP and PCT levels were measured on postoperative days (POD) one, three, five, and seven, and compared among patients without infections, those with bloodstream infections, and those with other infections (urinary, bronchoalveolar lavage, or drain). Multivariate logistic regression assessed the impact of PCT and CRP trends on short- and long-term mortality.

Results: Among 216 LDLT patients, 122 (56%) were analyzed after applying exclusion criteria. In patients without infections, median CRP and PCT levels were elevated on days one (CRP: 19, interquartile range (IQR): 11-29; PCT: 3, IQR: 1-13) and three (CRP: 18, IQR: 7-30; PCT: 2, IQR: 1-9) but decreased by days five (CRP: 10, IQR: 5-16; PCT: 1, IQR: 0.2-3) and seven (CRP: 10, IQR: 3-17; PCT: 0.7, IQR: 0.3-3). No absolute values of CRP or PCT effectively diagnosed infections. Patients with a decreasing trend in CRP from POD three to five to seven had survival rates of 90%, 86%, and 85% at 30, 90, and 600 days, respectively; in contrast, those with an increasing CRP trend had lower survival rates of 78%, 71%, and 40%, respectively. Similarly, a decline in PCT was associated with 30-, 90-, and 600-day survival rates of 90%, 85%, and 84%, while increasing PCT correlated with significantly lower rates of 75%, 70%, and 30%, respectively.

Conclusion: While the absolute values of CRP and PCT were not diagnostic for infection, an increase in these biomarkers from days three to five to seven predicted significantly higher short- and long-term mortality in LDLT recipients.

## Introduction

Bacterial sepsis following liver transplantation (LT) remains a significant cause of morbidity and mortality, with reported incidence ranging from 20-39% [1-5]. Early bacterial sepsis has a detrimental impact on long-term graft function and patient survival [6]. In the immediate postoperative period, liver transplant recipients often exhibit systemic inflammatory response syndrome (SIRS) triggered by reperfusion injury or surgical stress. This can mimic classical signs of sepsis such as fever, tachycardia, tachypnea, hypotension, and abnormal leukocyte counts, making the diagnosis challenging. Conversely, impaired hepatic function leads to reduced complement and cytokine production and Kupffer cell dysfunction, which can blunt or obscure the clinical and laboratory manifestations of sepsis [1,3,4]. These opposing processes frequently delay diagnosis and initiation of targeted antimicrobial therapy. The importance of early recognition and prompt empirical antibiotic treatment to mitigate sepsis-related mortality cannot be overstated [1-5].

Conventional markers, such as leukocyte count and C-reactive protein (CRP), lack sufficient specificity and sensitivity to distinguish infections from sterile inflammation due to surgical trauma or systemic stress. Procalcitonin (PCT), a 116-amino acid precursor of calcitonin, has emerged as a promising biomarker in this context. PCT is normally secreted by parafollicular C cells of the thyroid, as well as by lung, liver, and intestinal cells, and plays a role in calcium homeostasis. The first report of elevated PCT levels during bacterial sepsis was by Assicot et al. [7]. The calcitonin I (CALC-1) gene on chromosome 11 is thought to be induced in extra-thyroidal tissues by bacterial lipopolysaccharides and pro-inflammatory cytokines. PCT becomes detectable within two to four hours of a bacterial insult, peaks at 12-24 hours, and has a half-life of approximately 26-30 hours [8]. In healthy individuals, serum PCT levels are negligible (<0.5 ng/mL). Unlike CRP, PCT is not enzymatically degraded and remains stable in circulation. Importantly, PCT levels tend to normalize more rapidly than CRP in response to appropriate antimicrobial therapy, and are suppressed by interferon-γ during viral infections, potentially aiding in differentiating bacterial from viral etiologies [9].

Several studies have evaluated the utility of CRP and PCT in detecting sepsis after LT [10]. Moderate correlations have been observed between these markers and vasopressor requirement, mechanical ventilation, blood product transfusion, and operative duration, likely reflecting non-specific cytokine release. Immunosuppressive regimens, including anti-thymocyte globulin, corticosteroids, and calcineurin inhibitors, as well as underlying alcoholic liver disease, can also elevate CRP and PCT levels in the absence of infection [11,12]. Consequently, the diagnostic value of PCT in LT recipients has been inconsistent, with most data derived from deceased donor LT cohorts failing to demonstrate clear predictive utility for clinically significant infections [13].

Against this background, we conducted a study to compare the diagnostic accuracy of PCT and CRP in predicting postoperative infectious complications and mortality in the setting of live donor liver transplantation (LDLT).

## Materials and methods

Study design and participants

This prospective observational study was conducted at the Department of Solid Organ Transplantation, Amrita Institute of Medical Sciences and Research Center, India. All adult patients (≥18 years) who underwent LDLT between January 2021 and December 2022 were screened for inclusion.

Eligibility and exclusion criteria 

Inclusion Criteria

Adult recipients (≥18 years) who underwent living donor liver transplantation using the right lobe between January 2021 and December 2022.

Exclusion Criteria

Patients were excluded if they had any of the following characteristics: a culture-proven infection within two weeks before transplantation; Child-Pugh class A or B cirrhosis undergoing LDLT for hepatocellular carcinoma (HCC), since this group represents a relatively well-preserved cohort and would introduce heterogeneity, with only Child-Pugh class C cirrhosis patients included in the HCC subgroup; ABO-incompatible grafts, because these recipients have a higher infection risk due to the preoperative desensitisation protocol; and LDLT procedures using left lobes or left lateral lobes, which were omitted to avoid heterogeneity.

The study was approved by the Institutional Ethics Committee for Human Research and adhered to the principles of the Declaration of Helsinki and the International Conference on Harmonisation-Good Clinical Practice (ICH-GCP) guidelines.

Objectives

The primary objective of this study was to evaluate the diagnostic accuracy of C-reactive protein (CRP) and procalcitonin (PCT) in identifying bloodstream or localized infections-urinary tract infections, drain-associated infections, and bronchoalveolar lavage (BAL) infections-during the initial post-transplant period, specifically on days three, five, and seven. Secondary objectives included establishing optimal cut-off values for CRP and PCT in diagnosing infections, as well as assessing the prognostic value of these biomarkers for predicting 30-day, 90-day, and 24-month mortality.

Surgical and postoperative protocol

All recipients underwent right lobe LDLT. Standard intraoperative monitoring included central venous and radial arterial catheter insertion. All patients were shifted to the ICU postoperatively. Extubation was typically performed on postoperative day (POD) one, unless contraindicated. Routine Doppler ultrasonography was performed daily in the ICU to assess graft vascularity.

All patients received standard immunosuppression with tacrolimus, mycophenolate mofetil, and corticosteroids. Perioperative antibiotic prophylaxis consisted of piperacillin-tazobactam for three days. In patients with prior culture positivity (who were excluded from this study), targeted antibiotics were administered preoperatively. On POD three, cultures were obtained from peripheral and central blood, urine, and surgical drains. Invasive lines and catheters were removed by POD three and four unless clinically contraindicated. Drain removal was at the discretion of the operating surgeon. Apart from routine POD three cultures, additional cultures were obtained when clinically indicated (e.g., new fevers, organ dysfunction, collections). Any intra-abdominal collection was drained and sent for culture. BAL was performed in patients with ventilator dependence, reintubation, or new radiological infiltrates.

Infection diagnosis criteria

Infections were defined by positive microbiological cultures and clinical evidence of infection, based on Sepsis-3 criteria (≥2-point increase in Sequential Organ Failure Assessment (SOFA) score) [14].

We analyzed the prognostic significance of absolute values of CRP and PCT on the pre-specified postoperative days one, three, five, and seven. Additionally, we examined the importance of trends in CRP and PCT values, expressed as absolute increases or decreases from one day to the next.

Biomarker sampling and analysis

Routine laboratory parameters (CBC, liver and renal function tests, and coagulation profile) were obtained on the day of transplantation. CRP and PCT levels were measured on POD one, three, five, and seven, based on the known half-life of PCT (26-30 hours). Additional measurements were taken if there was a suspected infection or clinical deterioration. Procalcitonin (PCT) was measured using the Elecsys® BRAHMS PCT assay (Roche Diagnostics, Mannheim, Germany) based on electrochemiluminescence immunoassay (ECLIA); measuring range: 0.02-100 µg/L; functional sensitivity: 0.06 µg/L. CRP was measured using immune-turbidimetric analysis on a Beckman Coulter AU analyzer (Beckman Coulter Inc., Miami, USA). 

A standardized data collection form was used to capture clinical data, biochemical values, microbiological results, and SOFA score. Adjudication was by an infectious disease specialist.

Statistical analysis

The patients were analyzed into three separate groups: those without infection, those with bloodstream infection, and those with any infection other than blood (urine, BAL, or drain). All continuous variables were presented as means with standard deviations. All categorical variables were presented as frequencies with percentages. To test the statistical significance of the difference in the infection rates and non-infectious complications, chi-square and Fisher's exact tests were applied depending on the magnitude of the expected frequencies. An independent samples t-test/Kruskal-Wallis rank sum test was used to study the statistical significance of the difference in the mean values of all continuous variables between groups. Univariate logistic regression was applied to predict 30-day mortality. Stepwise variable selection was used to create multivariate logistic regression models for the influence of CRP and PCT on 30-day mortality. Patient survival was estimated by the Kaplan-Meier technique, and the difference in survival between the groups was tested for its statistical significance using the log-rank test. The univariable and multivariable analyses for 30-day mortality were repeated for long-term survival (90 days and 600 days) using Cox regression. The receiver operating characteristic (ROC) curves were used to visualize the trade-off between sensitivity and specificity of CRP and PCT. The Youden index was used to identify the potential for a good cut-off point. A P-value smaller than 0.05 was considered to be statistically significant.

## Results

**Figure 1:**
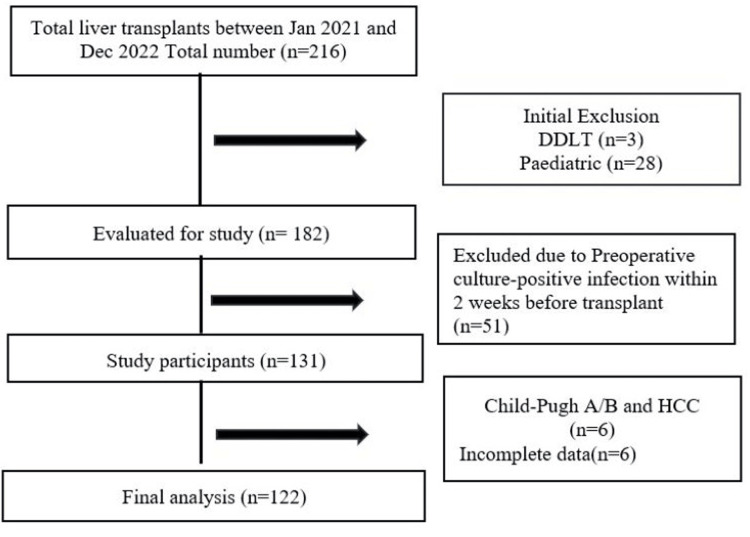
Study profile. HCC: hepatocellular carcinoma, DDLT: deceased donor liver transplants.

A total of 216 patients underwent LDLT during the study period, of which 94 (44%) were excluded: 51 (24%) due to preoperative culture positivity, 28 (13%) pediatric, 3 (1%) deceased donor transplants, 6 (3%) with Child-Pugh A/B and HCC, and 6 (3%) due to incomplete data. The final cohort comprised 122 patients (56%). Chronic liver disease due to alcohol (32%, n=39) and metabolic-associated steatotic liver disease (31%, n=38) were the leading etiologies (Figure 1).

Baseline demographics are presented in Table 1. Of the 122 patients, only 41 (33.6%) were discharged without any infectious complications. Bloodstream infections occurred in 45 patients (36.8%), while 15 (12.3%), 9 (7.3%), and 7 (5.7%) had culture-positive infections from urine, BAL, and surgical drains, respectively. Five patients (4%) had infections at multiple sites. Baseline characteristics, including age, model for end-stage liver disease (MELD) score, graft-to-recipient weight ratio, etiology, leukocyte and platelet counts, and early liver function parameters (aspartate aminotransferase (AST), alanine transaminase (ALT), international normalized ratio (INR)), were comparable across the three groups: no infection (n=41), bloodstream infection (n=45), and other infections (n=36).

**Table 1:** Baseline characteristics of patients with bloodstream infection, without any infection, and with infection at other sites (bronchoalveolar lavage, drain, or urine). MELD: model for end-stage liver disease, GRWR: graft recipient weight ratio, MASLD: metabolic dysfunction-associated steatotic liver disease, ALF: acute liver failure, HCC: hepatocellular carcinoma, HBV: hepatitis B virus, HCV: hepatitis C virus, WBC: white blood cell, INR: international normalized ratio, ALT: alanine transaminase, AST: aspartate aminotransferase. ^a^Median (interquartile range (IQR)); n (%) 51 (28, 89). ^b^Kruskal-Wallis's rank sum test; Pearson's chi-squared test.

Characteristic	Overall, N=122^a^	No infection, N=41^a^	BSI, N=45^a^	Other infections, N=36^a^	p-value^b^
Age^a^	49 (34, 55)	49 (41, 54)	45 (23, 52)	51 (43, 57)	0.031
Gender					0.4
Female	20 (16%)	4 (9.8%)	9 (20%)	7 (19%)	
Male	102 (84%)	37 (90%)	36 (80%)	29 (81%)	
MELD	24 (19, 29)	23 (18, 29)	25 (19, 31)	22 (19, 28)	0.6
GRWR	0.92 (0.76, 1.20)	0.92 (0.76, 1.18)	0.90 (0.75, 1.14)	1.05 (0.80, 1.30)	0.5
Etiology					
Autoimmune	20 (16%)	1 (2.4%)	10 (22%)	9 (25%)	
MASLD	38 (31%)	13 (32%)	11 (24%)	14 (39%)	
Ethanol	39 (32%)	15 (37%)	14 (31%)	10 (28%)	
ALF	9 (7.4%)	4 (9.8%)	4 (8.9%)	1 (2.8%)	
HCC	8 (6.6%)	4 (9.8%)	3 (6.7%)	1 (2.8%)	
HBV	5 (4.1%)	3 (7.3%)	1 (2.2%)	1 (2.8%)	
HCV	2 (1.6%)	1 (2.4%)	1 (2.2%)	0 (0%)	
WBC day one	9.3 (6.6, 13.3)	8.6 (6.2, 11.8)	9.7 (7.0, 15.8)	8.9 (6.5, 11.8)	0.5
WBC day three	8.7 (6.1, 11.9)	7.7 (6.2, 11.5)	8.7 (6.4, 12.1)	9.3 (5.3, 12.0)	0.6
WBC day five	8.2 (6.4, 11.3)	8.2 (6.5, 10.9)	8.7 (6.4, 12.9)	7.9 (6.3, 11.2)	0.9
WBC day seven	9.3 (6.7, 14.1)	8.9 (6.5, 10.5)	10.9 (7.7, 15.4)	9.6 (6.6, 14.5)	0.2
Platelet day one	80 (44, 120)	91 (53, 152)	73 (42, 114)	77 (47, 106)	0.5
Platelet day three	71 (36, 123)	71 (37, 133)	59 (34, 105)	74 (40, 115)	0.6
Platelet day five	70 (40, 113)	80 (45, 132)	69 (36, 105)	76 (44, 126)	0.5
Platelet day seven	87 (53, 119)	90 (50, 119)	86 (59, 118)	83 (54, 116)	>0.9
INR day one	2.10 (1.59, 2.84)	1.99 (1.10, 2.77)	2.25 (1.73, 3.01)	2.10 (1.71, 2.71)	0.078
INR day three	1.78 (1.25, 2.34)	1.67 (1.09, 2.22)	1.78 (1.41, 2.41)	1.81 (1.30, 2.29)	0.2
INR day five	1.40 (1.06, 1.81)	1.12 (1.01, 1.60)	1.44 (1.22, 1.95)	1.47 (1.10, 1.83)	0.038
INR day seven	1.21 (1.01, 1.57)	1.12 (0.97, 1.53)	1.30 (1.09, 1.89)	1.09 (1.01, 1.45)	0.094
ALT day one	156 (98, 314)	133 (93, 266)	195 (124, 413)	155 (80, 307)	0.2
ALT day three	135 (67, 298)	123 (57, 266)	154 (74, 408)	111 (71, 273)	0.5
ALT day five	89 (55, 199)	84 (57, 198)	121 (55, 242)	76 (48, 172)	0.5
ALT day seven	79 (45, 172)	71 (41, 146)	88 (62, 284)	72 (41, 150)	0.2
AST day one	139 (60, 256)	109 (60, 241)	187 (73, 319)	118 (51, 213)	0.2
AST day three	70 (45, 175)	64 (37, 163)	84 (54, 250)	56 (42, 130)	0.15
AST day five	53 (32, 88)	42 (26, 77)	64 (42, 169)	50 (32, 92)	0.047
AST day seven	51 (28, 89)	39 (24, 83)	69 (45, 123)	54 (27, 85)	0.094

**Figure 2:**
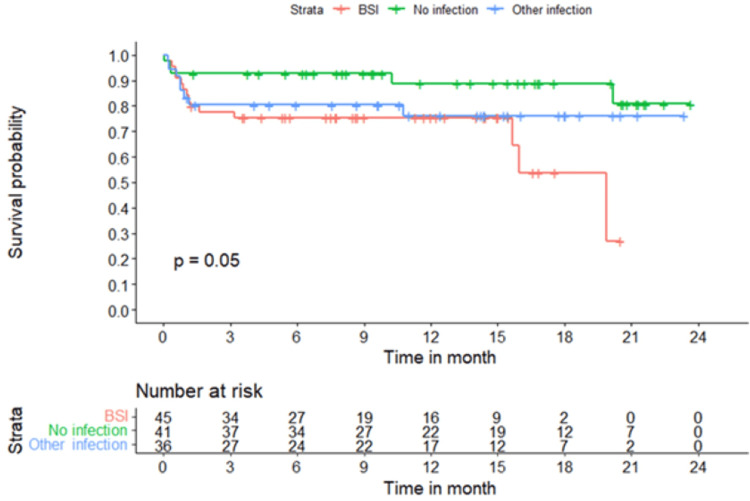
Thirty-day mortality: Kaplan-Meier survival curves comparing patients with no infection, bloodstream infection (BSI), and other infections. 30-day mortality was lowest in patients with no infections and highest in those with bloodstream infections (BSI).

Table 2 presents the postoperative details in the first week for the three groups: those without infection, with bloodstream infection, and with any infection (urine, BAL, or drain). ICU and hospital stay durations, as well as mortality rates, were not statistically different between the groups. Over a median follow-up of 313 days (IQR: 134.8-485), 27 patients (22%) died, including 21 within the first 30 days. The 30-day mortality rates were 24% (n=11) in patients with bloodstream infections, 19% (n=7) in those with other infections, and 7.3% (n=3) in those without infection (p=0.21). Although long-term mortality was lower among patients without infections during their index admission (12%, n=5) than in those with bloodstream (31%, n=14) or other infections (22%, n=8), the difference was not statistically significant (Table 2, Figure 2).

**Table 2:** Serial CRP and PCT values within the first week, ICU and hospital stay, and mortality in patients without infection, with bloodstream infection, and with infection at other sites (bronchoalveolar lavage, drain, or urine). CRP: C-reactive protein, PCT: procalcitonin, ICU: intensive care unit. ^a^Median (IQR); n (%). ^b^Kruskal-Wallis rank sum test; Pearson's chi-squared test.

Characteristic	Overall, N=122^a^	No infection, N=41^a^	BSI, N=45^a^	Other infections, N=36^a^	p-value^b^
CRP day one^a^	23 (12, 56)	19 (11, 29)	30 (12, 70)	25 (15, 82)	0.058
CRP day three^a^	21 (12, 44)	18 (7, 30)	25 (17, 46)	27 (13, 66)	0.011
CRP day five^a^	14 (8, 33)	10 (5, 16)	18 (9, 45)	23 (11, 51)	<0.001
CRP day seven^a^	14 (6, 34)	10 (3, 17)	16 (9, 54)	20 (7, 45)	0.013
PCT day one^a^	3 (1, 13)	3 (1, 13)	3 (1, 12)	3 (1, 20)	0.8
PCT day three^a^	2 (1, 9)	2 (0.2, 7)	4 (1, 9)	2 (1, 13)	0.5
PCT day five^a^	1.2 (0.4, 4.5)	1.1 (0.2, 3.1)	1.4 (0.4, 4.5)	1.2 (0.5, 6.3)	0.5
PCT day seven^a^	0.8 (0.3, 2.5)	0.7 (0.3, 3.1)	0.9 (0.4, 4.1)	0.9 (0.3, 2.0)	0.7
ICU stay (days)^a^	10 (7, 14)	9 (7, 11)	9 (7, 16)	11 (8, 14)	0.15
Hospital stays (days)^a^	21 (16, 27)	20 (15, 24)	19 (16, 28)	24 (19, 27)	0.4
30-day mortality (n (%))	21 (17%)	3 (7.3%)	11 (24%)	7 (19%)	0.10
Long-term mortality (n (%))	27 (22%)	5 (12%)	14 (31%)	8 (22%)	0.11

Postoperative CRP and procalcitonin (PCT) trends

**Figure 3:**
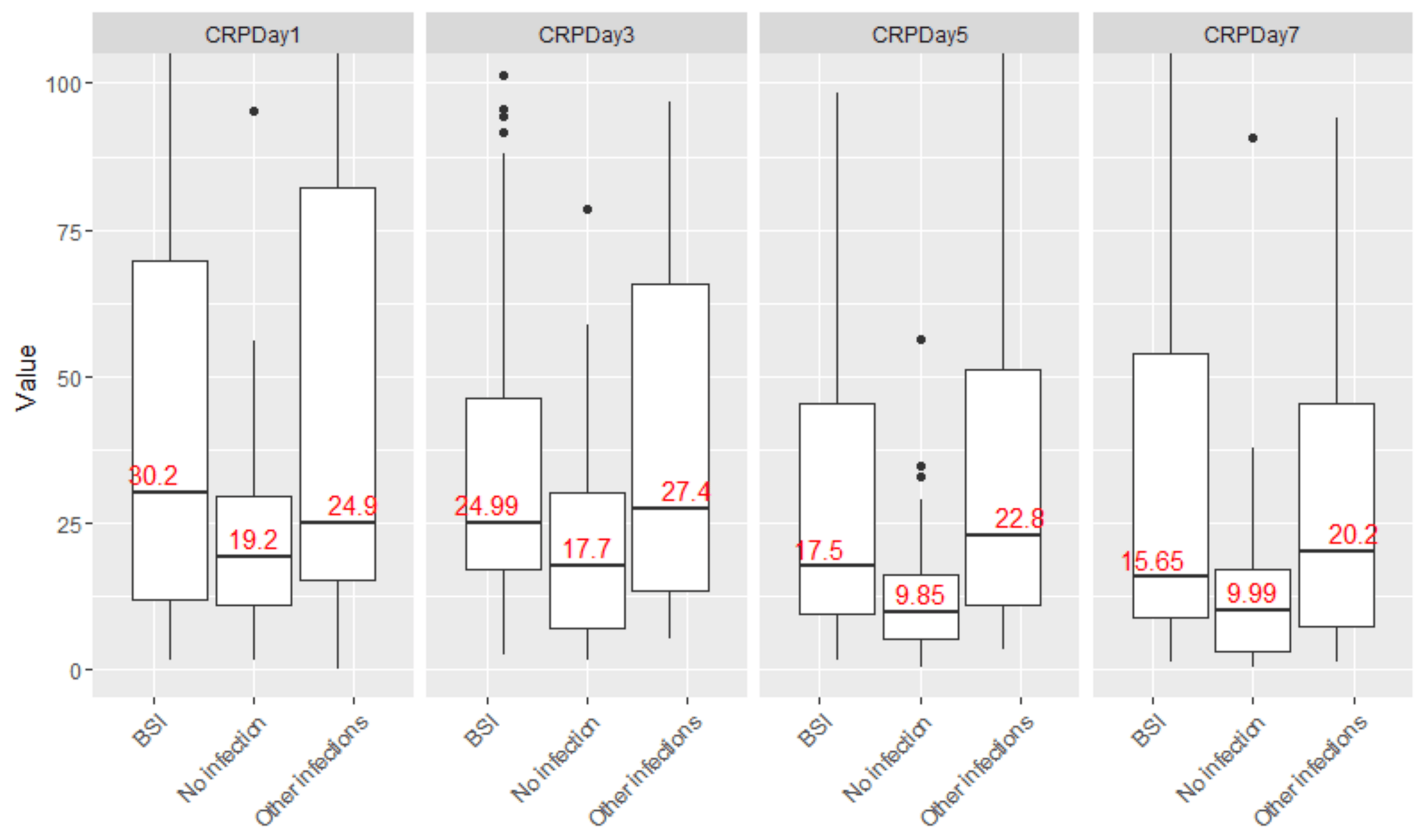
CRP values in postoperative periods in patients with bloodstream infection, no infection, or other infections. CRP values on postoperative days one, three, five, and seven in patients with bloodstream infection, no infection, or other infection were elevated across all three groups, with significant overlap between patients with and without infection. BSI: bloodstream infection; CRP: C-reactive protein.

**Figure 4:**
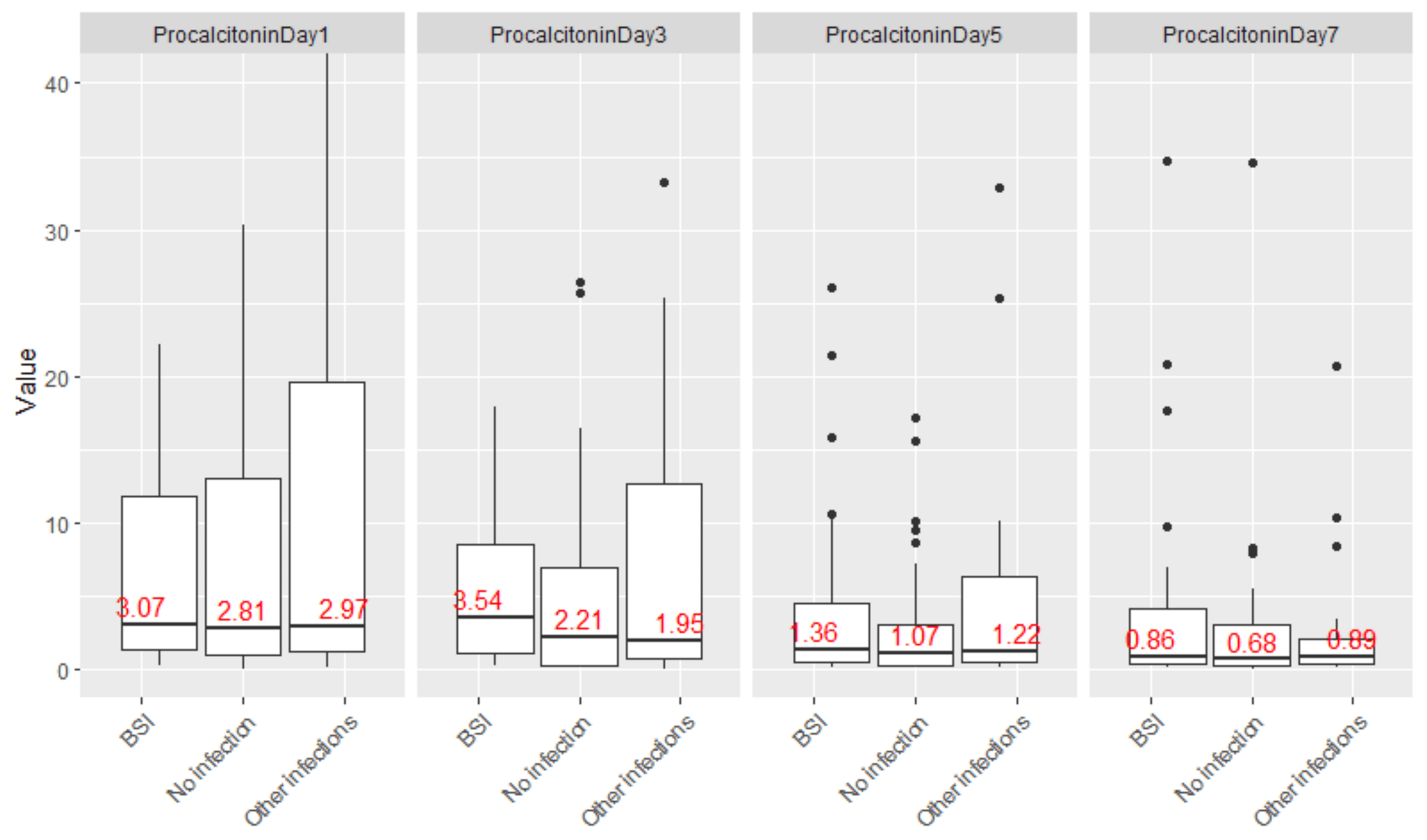
PCT values on postoperative periods in patients with bloodstream infection, no infection, or other infections. PCT values on postoperative days one, three, five, and seven were elevated across all groups, with significant overlap between patients with and without infection. PCT: procalcitonin; BSI: bloodstream infection.

CRP and PCT values on postoperative days (POD) one, three, five, and seven are summarized in Table 2 and visualized in Figures 3, 4.

Both biomarkers were markedly elevated post-transplantation, regardless of infection status. Among patients without infection (n=41), median CRP values were 19, 18, 10, and 10 mg/L on PODs one, three, five, and seven, respectively. In the bloodstream infection group (n=45), CRP medians were 30, 25, 18, and 16 mg/L. For other infections (n=36), values were 25, 27, 23, and 20 mg/L, respectively. While median CRP values were higher in patients with infections, there was significant overlap between groups, precluding the identification of a reliable diagnostic threshold.

Median PCT levels followed a similar trend. In patients without infection, values declined from 3 µg/L on POD one to 0.7 µg/L by POD seven. In those with bloodstream infections, the median PCT values were 3, 4, 1.4, and 0.9 µg/L, while in other infections, the corresponding values were 3, 2, 1.2, and 0.9 µg/L. No statistically significant difference in PCT values was observed between groups on any postoperative day.

**Figure 5:**
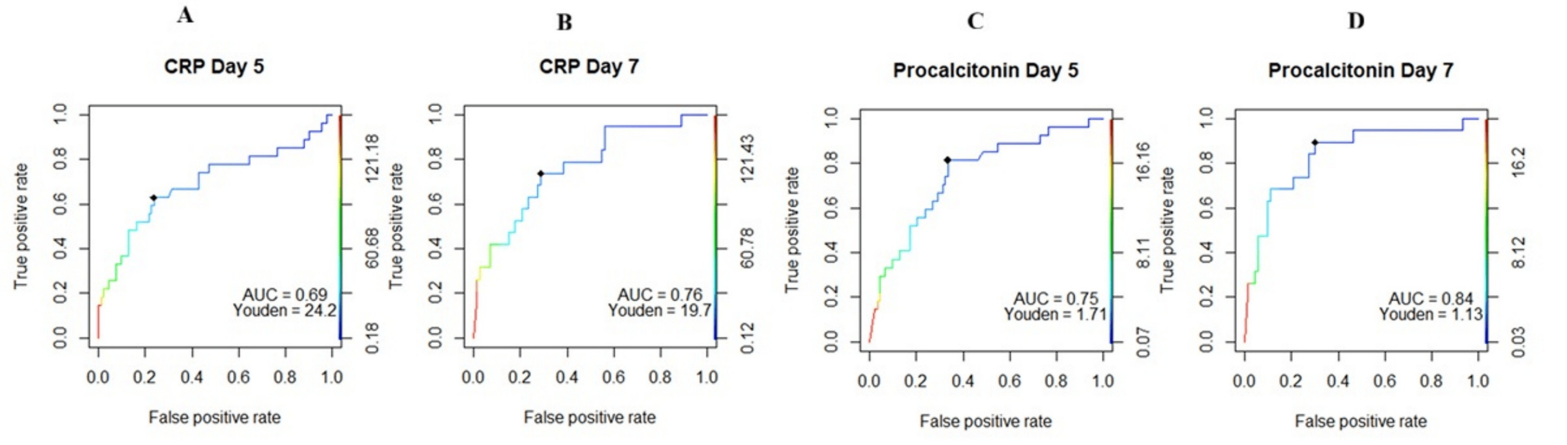
ROC analysis for CRP and PCT on postoperative days three and five. (A) ROC analysis for CRP on day five; (B) ROC analysis for CRP on day seven; (C) ROC analysis for PCT on day five; and (D) ROC analysis for PCT on day seven. ROC: receiver operating characteristic; CRP: C-reactive protein; PCT: procalcitonin; AUC: area under the curve.

ROC curve analysis (Figure 5) showed that CRP on POD seven had the highest predictive accuracy for diagnosing culture-positive infections (area under the curve (AUC): 0.76, Youden index: 19.7, sensitivity: 0.74, specificity: 0.71). Procalcitonin (PCT) levels on day seven exhibited enhanced predictive capability, with an area under the curve (AUC) of 0.84 and a Youden index of 1.13, demonstrating a sensitivity of 0.89 and specificity of 0.70. In comparison, PCT levels on day five had a lower AUC of 0.75 and a Youden index of 1.71, with sensitivity and specificity values of 0.81 and 0.66, respectively.

Prognostic value of biomarker trends

**Figure 6:**
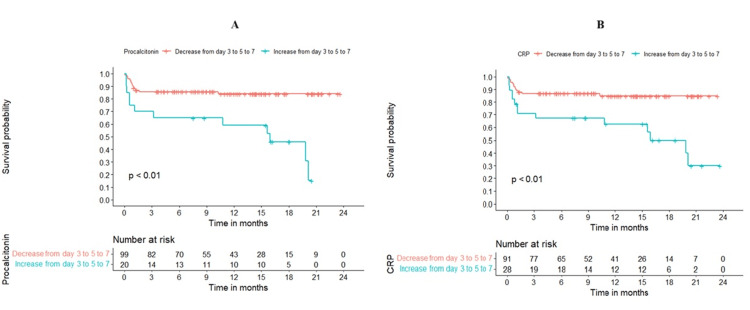
Survival curves for different trends of PCT (A) and CRP (B). PCT: procalcitonin; CRP: C-reactive protein.

Although absolute CRP and PCT values were not individually predictive of infection, their postoperative trends were significantly associated with mortality outcomes (Figures 6A, 6B). Patients demonstrating a downward trend in CRP from POD three to POD seven had 30-day, 90-day, and 600-day survival rates of 90%, 86.8%, and 85%, respectively. In contrast, those with an increasing CRP trend had corresponding survival rates of 78.6%, 71.1%, and 40%. Similarly, patients with a decline in PCT from POD three to POD seven had survival rates of 90%, 85.8%, and 84.1%. In contrast, increasing PCT was associated with significantly lower survival: 75% (30-day), 70% (90-day), and 30.6% (600-day).

In the multivariate analysis presented in Table 3, several significant predictors of 30-day mortality were identified: an increase in CRP from day three (odds ratio (OR) 1.6), an increase in PCT from day three (OR 1.4), positivity of bronchoalveolar lavage (BAL) cultures (OR 1.5), and female gender (OR 1.1).

**Table 3:** Univariate and multivariate logistic regression model for 30-day mortality. PCT: procalcitonin, CRP: C-reactive protein, MELD: model for end-stage liver disease, GRWR: graft recipient weight ratio, MASLD: metabolic dysfunction-associated steatotic liver disease, ALD: alcoholic liver disease, ALF: acute liver failure, HCC: hepatocellular carcinoma, HBV: hepatitis B virus, HCV: hepatitis C virus, BAL: bronchoalveolar lavage, OR: odds ratio, CI: confidence interval.

Characteristic	Univariate			Multivariate		
	Log (OR)	95% CI	p-value	Log (OR)	95% CI	p-value
PCT						
Decrease from day three to five	-	-		-	-	
Increase from day three to five	2.4	1.3, 3.5	<0.001	1.6	0.17,3.0	0.029
CRP						
Decrease from day three to five	-	-		-	-	
Increase from day three to five	2.2	1.2, 3.3	<0.001	1.4	0.01, 2.7	0.04
Gender						
Male	-	-		-	-	
Female	1.2	0.11, 2.3	0.027	1.1	-0.06, 2.2	0.054
Age	-0.02	-0.05, 0.01	0.13			
MELD	0.03	-0.03, 0.09	0.3			
GRWR	-0.43	-1.9, 0.37	0.5			
Etiology						
MASLD	-	-				
ALD	0.37	-0.87, 1.7	0.6			
ALF	0.63	-1.4, 2.4	0.5			
HBV and HCV	1.6	-0.24, 3.4	0.076			
HCC	-16	-498, 40	>0.9			
Autoimmune	0.44	-1.1, 1.9	0.5			
Blood culture positive	0.77	-0.18, 1.7	0.11			
Urine culture positive	0.32	-0.74, 1.3	0.5			
BAL culture positive	1.6	0.52, 2.7	0.003	1.5	0.41, 2.7	0.006
Drain culture positive	1.2	-0.27, 2.5	0.091			
Any culture positive	0.66	-0.33, 1.8	0.2			

Furthermore, the multivariate Cox regression model for long-term mortality, as shown in Table 4, identified the following significant predictors: a rise in CRP from day three (HR 3.35), a rise in PCT from day three (HR 2.95), gender (HR 2.57), positivity of blood cultures (HR 2.33), and positivity of BAL cultures (HR 3.01).

**Table 4:** Univariate and multivariate Cox regression for long-term mortality. PCT: procalcitonin, CRP: C-reactive protein, MELD: model for end-stage liver disease, GRWR: graft recipient weight ratio, MASLD: metabolic dysfunction-associated steatotic liver disease, ALD: alcoholic liver disease, ALF: acute liver failure, HCC: hepatocellular carcinoma, HBV: hepatitis B virus, HCV: hepatitis C virus; HR: hazard ratio; CI: confidence interval.

Characteristic	Univariate			Multivariate		
	HR	95% CI	p-value	HR	95% CI	p-value
PCT						
Decrease from day three to five	-	-		-	-	
Increase from day three to five	4.28	2.00, 9.19	<0.001	3.35	1.48, 7.57	0.004
CRP						
Decrease from day three to five	-	-		-	-	
Increase from day three to five	3.72	1.74, 7.93	<0.001	2.95	1.30, 6.69	0.013
Gender						
Male	-	-		-	-	
Female	3.19	1.43, 7.12	0.005	2.57	1.13, 5.84	0.024
Age	0.98	0.96, 1.00	0.087			
MELD	1.03	0.98, 1.08	0.2			
GRWR	0.57	0.20, 1.64	0.3			
Etiology						
MASLD	-	-				
ALD	1.78	0.60, 5.33	0.3			
ALF	5.50	1.58, 19.1	0.007			
HBV and HCV	3.05	0.73, 12.8	0.13			
HCC	0.87	0.10, 7.46	0.9			
Autoimmune hepatitis	1.63	0.43, 6.10	0.5			
Blood culture positive	2.29	1.05, 4.97	0.036	2.33	1.06, 5.09	0.035
Urine culture positive	0.88	0.37, 2.08	0.8			
Drain culture positive	2.00	0.69, 5.78	0.2			
BAL culture positive	3.35	1.48, 7.58	0.004	3.01	1.31, 6.95	0.010
All culture positive	1.63	0.72, 3.67	0.2			

Table 5 details the organisms isolated in culture, with *Klebsiella pneumoniae* being the most commonly identified organism from blood cultures 49% (n=22). Other frequently isolated organisms included coagulase-negative staphylococcus 8.9% (n=4), Enterococci 6.7% (n=3), and *Pseudomonas aeruginosa* 6.7% (n=3). Additionally, *Klebsiella pneumoniae* and Enterococci were the predominant organisms found in urine, drain, and BAL cultures.

**Table 5:** Organisms grown in blood culture. ^a^n (%).

Characteristic	N=45^a^
Organism	
Klebsiella pneumoniae	22 (49%)
Coagulase-negative staphylococcus	4 (8.9%)
Enterococcus	3 (6.7%)
Pseudomonas aeruginosa	3 (6.7%)
Escherichia coli	2 (4.4%)
Elizabethkingia meningoseptica	2 (4.4%)
Staphylococcus aureus	2 (4.4%)
Acinetobacter baumannii	1 (2.2%)
Aerobic spore bearers	1 (2.2%)
Alpha hemolytic streptococci	1 (2.2%)
Burkholderia cepacia	1 (2.2%)
Gram-positive cocci	1 (2.2%)
Granulicatella adiacens	1 (2.2%)
Staphylococcus fumigatus	1 (2.2%)

## Discussion

In our study, we observed that both CRP and procalcitonin PCT exhibited elevated levels in the immediate post-transplant period. This nonspecific elevation of inflammatory markers during the postoperative period has been documented in previous research and does not necessarily correlate with poor prognosis [15-17]. Notably, PCT and CRP demonstrated comparably high values on postoperative days one, three, five, and seven, regardless of whether there was evidence of infection (positive blood, urine, drain, or bronchoalveolar lavage cultures) or not. While absolute values of CRP and PCT did not demonstrate diagnostic utility for infection, we identified that the trends of both markers over the initial week are predictive of mortality. In patients without infection, a decline in both CRP and PCT from day one through day seven was associated with improved survival rates. Conversely, a rise in these values from day three to day five to day seven was significantly predictive of both short-term and long-term mortality. Thus, rather than relying on a single measurement, serial monitoring of CRP and PCT levels during the first week may serve as valuable prognostic markers for predicting mortality risk. Rising trends in CRP or procalcitonin during this period warrant increased vigilance from the transplant physician regarding the possibility of infection. It may be necessary to adjust antibiotic therapy or conduct further investigations to rule out abdominal, chest, or urinary infections. A previous study from Germany indicated that a second peak in PCT during an uneventful post-transplant setting was significant for patients with severe bacterial or fungal sepsis and graft dysfunction, highlighting the importance of monitoring inflammatory markers [16].

The underlying pathophysiology for the initially elevated values of CRP and PCT following transplant remains incompletely understood; however, it has been hypothesized that these elevations may stem from surgical stress, production from splanchnic circulation, blood product transfusions, and the initiation of immunosuppression [18]. It is plausible that the typical reference ranges of PCT and CRP may be elevated in the post-transplant period compared to pre-surgical values. Establishing an adjusted normal range for post-transplant patients could facilitate the early detection of variations associated with sepsis. Our results align with findings from Eyraud et al., which demonstrated that initial PCT values lack diagnostic or prognostic significance. In our analysis, the receiver operating characteristic (ROC) curve revealed that a CRP value of 19.7 on day seven exhibited a sensitivity of 0.74 and specificity of 0.71 for diagnosing infection, while a PCT value of 1.13 on day seven demonstrated a higher sensitivity of 0.89 but with similar specificity.

Numerous studies have pointed to the superior diagnostic accuracy of PCT compared to CRP for bacterial and fungal infections and sepsis across various patient populations [12,19,20]. Although comparisons between PCT and CRP in the context of liver transplantation have been conducted, many studies have focused primarily on deceased donor liver transplant recipients. The kinetics and induction of procalcitonin (PCT) and C-reactive protein (CRP) after various surgical procedures have been explored by several authors. They have reported that PCT levels rise much less than CRP and have a shorter period of induction. A limitation in the diagnostic accuracy of PCT was noted by Zazula et al., who found that an ATG-based immunosuppressive regimen could stimulate PCT synthesis [12]. However, this limitation did not affect our study, as we do not routinely use ATG in our immunosuppressive protocol. Nonetheless, the low sensitivity of both CRP and PCT indicates that normal values do not necessarily rule out infections, and clinicians should not be misled by these results.

We also noted a trend in CRP and PCT levels declining from postoperative day one to subsequent days in patients without sepsis. Interestingly, despite their relatively short half-lives--26 to 30 hours for CRP and approximately 18 hours for PCT--these values did not return to pre-transplant ranges until postoperative day seven, even among patients without culture-proven infections. This suggests that surgical stress and resultant inflammation may persist for up to a week following liver transplantation. Furthermore, we did not find statistically significant correlations between PCT and CRP and liver function tests, leucocyte count, or platelet levels. This indicates that both PCT and CRP may not be influenced by fluctuations in liver function, although substantial inter-patient variability was observed.

In our multivariate analysis for 30-day mortality and Cox regression analysis for long-term mortality, rising trends of PCT and CRP from day three, along with BAL culture positivity, emerged as significant predictive factors. Interestingly, blood culture positivity was identified as a predictor of long-term but not 30-day mortality. Additionally, female gender was a significant factor for mortality, consistent with findings from other studies, likely due to higher immunological responses leading to chronic rejection, hormonal influences on liver regeneration, and a higher burden of comorbid conditions at the time of transplant [21,22]. While we cannot definitively claim that elevated CRP and PCT levels in the initial week are predictive of long-term mortality, it is plausible that they reflect the higher mortality observed during the first three months post-transplant, as evidenced by the steep decline in the Kaplan-Meier curve for long-term mortality. In the largest study to assess the role of PCT in the post-liver transplant setting, Vijayan et al. compared PCT to traditional markers such as CRP and leukocyte count. They found that peak PCT was not an independent predictor of sepsis or serious infections, while highlighting CRP as an independent risk factor for critical systemic infections. Additionally, they identified male sex, low BMI, acute liver failure, and prolonged cold ischemic time as independent risk factors for clinically significant infections [23].

Our study is a prospective analysis, with predefined protocols, of a homogeneous adult living LDLT population, examining the impact of rising trends in CRP and PCT on predicting short- and long-term mortality. However, several limitations must be acknowledged. Firstly, this study employed a single-center design, necessitating validation across additional institutions with larger patient cohorts. Secondly, although prospective in nature, several participants were excluded, primarily due to preoperative culture positivity. Additionally, six hepatocellular carcinoma patients with Child-Pugh class A or B were omitted; however, this small sample size of exclusion is unlikely to have significantly impacted the findings. Thirdly, the study did not include comparisons with other dynamic markers such as white cell count trends, neutrophil-to-lymphocyte ratios, or SOFA score trajectories. Fourthly, there exists an inherent risk of confounding, as increasing biomarker values of CRP and PCT may reflect general clinical decline due to graft dysfunction or non-infectious SIRS, rather than specifically predicting infection-related increment. A more detailed analysis of inflammatory markers may provide further insights into these dynamics.

## Conclusions

In this prospective observational study assessing the predictive utility of CRP and PCT following LDLT, non-specific elevations in both biomarkers were observed during the initial postoperative week, irrespective of infection status. Reliable diagnostic cut-off values for infection were not established prior to postoperative day seven. On day seven, a CRP concentration of 19.7 mg/L yielded a sensitivity of 0.74 and specificity of 0.7, whilst a PCT concentration of 1.13 ng/mL demonstrated higher sensitivity (0.89) with similar specificity. Crucially, dynamic trends in CRP and PCT throughout the first postoperative week provided greater clinical insight than absolute values alone. Increases in CRP and PCT from day three (HR 3.35 and HR 2.95, respectively), female sex (HR 2.57), positive blood cultures (HR 2.33), and positive BAL cultures (HR 3.01) emerged as significant predictors of 30-day, 90-day, and 600-day mortality. These results indicate that clinicians should prioritize the monitoring of upward CRP and PCT trajectories over isolated measurements to enhance the detection of infection and evaluate mortality risk following LDLT. An increasing trend between POD three and POD seven warrants a structured sepsis assessment and reconsideration of antimicrobial management. 
